# The encoded and expressed biosynthetic potential of Greenland Ice Sheet microbes

**DOI:** 10.3389/fmicb.2025.1620548

**Published:** 2025-07-31

**Authors:** Ate H. Jaarsma, Katie Sipes, Athanasios Zervas, Helen K. Feord, Francisco Campuzano Jiménez, Mariane S. Thøgersen, Liane G. Benning, Martyn Tranter, Alexandre M. Anesio

**Affiliations:** ^1^Department of Environmental Science, Aarhus University, Roskilde, Denmark; ^2^GFZ Helmholtz Centre for Geosciences, Potsdam, Germany; ^3^Department of Earth Sciences, Freie Universität Berlin, Berlin, Germany

**Keywords:** supraglacial habitats, biosynthetic gene clusters (BGCs), metatranscriptomics, microbial community ecology, Greenland Ice Sheet

## Abstract

Supraglacial habitats of the Greenland Ice Sheet (GrIS) harbor active microbial communities. Microbes produce a plethora of natural products, which hold great promise in biotechnology. Understudied environments such as the Greenland Ice Sheet are therefore of interest for the discovery of unknown biosynthetic gene clusters (BGCs) that encode these compounds. Though many applications of these natural products have been identified, little is known about their ecological function for the producer itself. Some hints exist toward roles in competition and environmental adaptation, yet confirmation of the expression of these BGCs in the natural environment is often lacking. Here, we investigated the expression of BGCs in supraglacial habitats of the GrIS. Using total RNA sequencing, we conducted a seasonal study to analyze metatranscriptomes of ice and cryoconite habitats over a 21-day period during the ablation season. Genome mining on metagenomic contigs identified BGCs within ice and cryoconite metagenomes, after which the metatranscriptomes were mapped to them. Our study identified a majority of previously unknown BGCs, 59% of which are actively expressed *in situ*, with relatively stable expression levels throughout the melting season. The 10 most highly expressed BGCs in ice were of eukaryotic origin, whereas in cryoconite, the 10 most highly expressed BGCs were prokaryote-derived. Among these was biosynthetic machinery for the production of carotenoids, terpenes, beta-lactones, and modified peptides, and their producers are likely ecosystem engineers of the supraglacial habitats, such as glacier ice or snow algae, and cyanobacteria. These findings highlight the significant, yet mostly unexplored, biosynthetic capabilities of GrIS supraglacial microbes, and suggest an active role of these BGCs in community ecology.

## 1 Introduction

Microbes produce a wide variety of chemical compounds, or natural products, that aid their survival (Gavriilidou et al., 2022). This chemical diversity is important for medicine and biotechnology; inspiring novel antibiotics (Clardy and Walsh, 2004), anticancer drugs (Cragg and Newman, 2013), and antivirals (Ma et al., 2020). The presence of biosynthetic gene clusters (BGCs) encoding these natural products can be predicted through genome mining of sequencing data (Medema et al., 2021), including environmental (meta)genomes.

The Greenland Ice Sheet (GrIS) is a biome that is driven by microbial activity in habitats such as the ice surface and cryoconite holes (Anesio et al., 2017). *Ancylonema* spp., eukaryotic glacier ice algae (Zygnematophyceae, Streptophyta), dominate the ice surface (Lutz et al., 2018), accelerating its melt by their dark pigmentation (Cook et al., 2020). Besides these algae, the supraglacial microbiome contains a diverse community of bacterial and fungal heterotrophs (Anesio et al., 2017). Cyanobacteria dominate the biomass in cryoconite sediment, which forms cryoconite holes by melting down into the ice (Cook et al., 2016). Common bacterial phyla in these supraglacial habitats are Pseudomonadota, Actinomycetota, and Bacteroidota (Jaarsma et al., 2023a).

Microorganisms living in extreme environments, such as the cryosphere, are under-explored sources of novel biosynthetic potential. Previous studies found unknown biosynthetic gene clusters in sequencing data from the European Alps, Arctic, Antarctic, and the “third pole,” the Tibetan plateau (Soldatou et al., 2019; Marcolefas et al., 2019; Waschulin et al., 2022; Liu et al., 2022; Medeiros et al., 2024, 2025; Rego et al., 2021; Turchetti et al., 2022; Busi et al., 2023; Geers et al., 2024). We previously found that isolate genomes and metagenome-assembled genomes (MAGs) from supraglacial habitats of the Greenland Ice Sheet harbor a wide range of genetically encoded chemical diversity, the majority of these natural products being thus far undescribed (Jaarsma et al., 2023b). This illustrates a large potential for discovering new compounds with novel applications, but also a large need for further characterization of many of the encoded natural products from these critically threatened habitats (Edwards et al., 2020). We found indications of potential survival mechanisms for the extreme conditions of the ice sheet, as these microbes seem to have the capacity to produce UV-blocking pigments, siderophores, and osmoprotectants, in addition to antimicrobial compounds. The ability to produce these compounds may aid survival under high UV radiation, nutrient limitation, freeze-thaw cycles, and microbial competition (Jaarsma et al., 2023b).

Our ability to speculate about the ecological functions of microbial natural products is limited by the lack of evidence showing that these gene clusters are expressed in their natural environment. Since the encoded biosynthetic potential is potentially linked to environmental adaptation (Jaarsma et al., 2023b), it can be hypothesized that part of it is likely used *in situ*. With this study we aimed to assess the active fraction of biosynthetic gene clusters in the supraglacial habitats of the Greenland Ice Sheet. Metatranscriptomes of ice and cryoconite habitats were sequenced from samples collected over 21 days during the ablation season. We performed genome mining on metagenomic contigs to identify BGCs within the ice and cryoconite metagenomes and mapped the metatranscriptomes to the identified BGCs to examine whether supraglacial microbes actively employ their biosynthetic potential in their natural environment.

## 2 Materials and methods

### 2.1 Study site and environmental context

**Figure 1:**
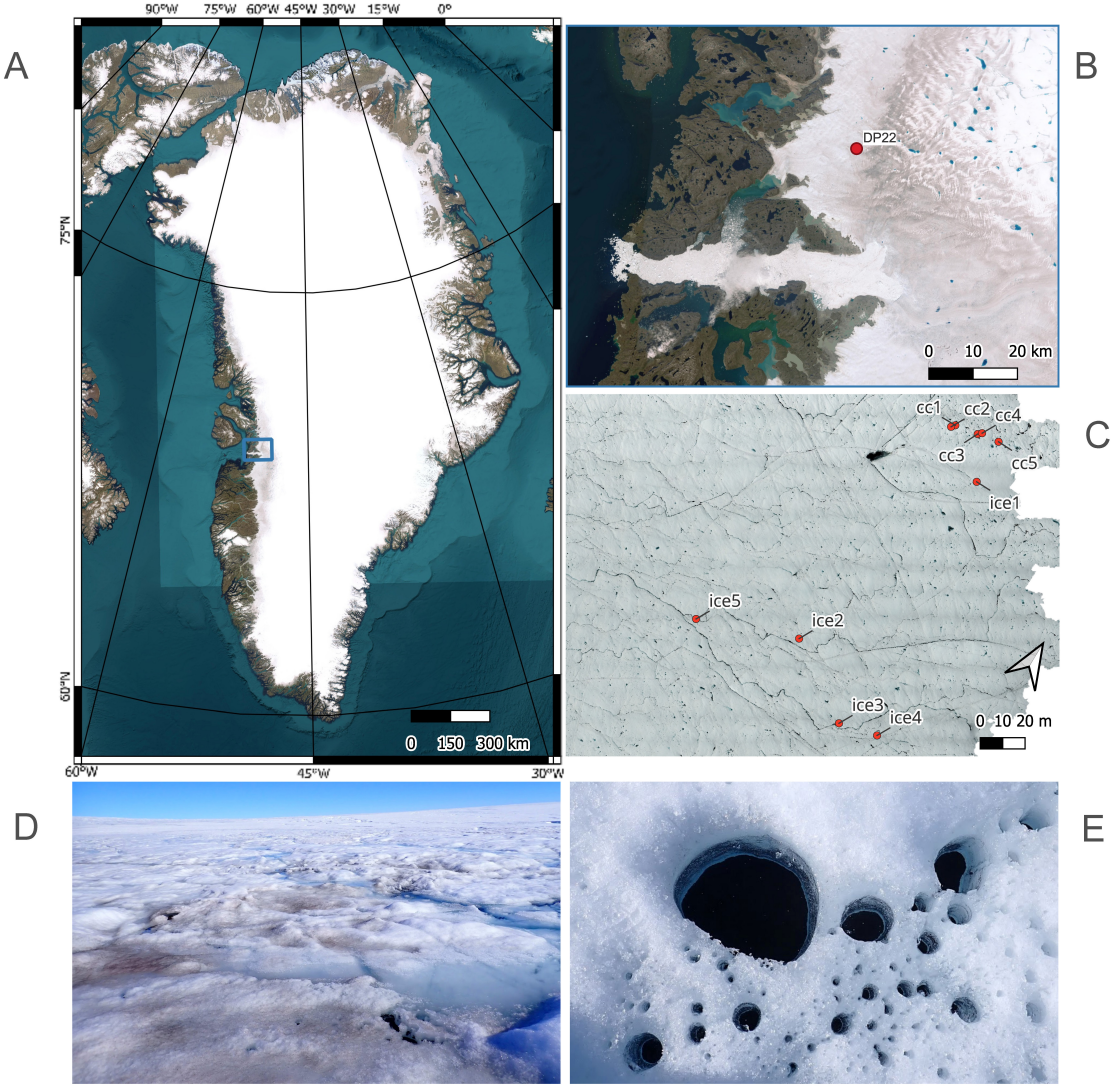
**(A)** The Greenland Ice Sheet, with fieldwork site **(B)**. **(C)** Selected cryoconite holes (cc 1–5) and ice sites (ice 1–5) for seasonal sampling. **(D)** Dark Surface Ice. **(E)** Cryoconite holes. Map layers were created using Esri, Maxar, Earthstar Geographics, and the GIS User Community.

The study site was located c. 12 km inland of the western margin of the Greenland Ice Sheet near Ilulissat (69.43 N, 49.86 W, 680 m asl). Five cryoconite holes and five 2-meter patches of bare ice were selected as sample sites (Figure 1), and sampled over 21 days between July 28 and August 18, 2022 (DOY 209–230). Biomass from the cryoconite holes and ice surface was collected at solar noon on seven sampling days, to minimize variation in solar zenith angle. The ice surface was snow-free from the start of the sampling period, but a snowfall event occurred on August 9 and the resulting snow cover remained until August 13. Between July 28 and August 14, the air temperature (at 2 m) ranged between -2.1 and 7.1°C (measured per minute, reported as hourly averages using HOBO 12-bit Temp/RH sensor). Precipitation totaled 19 mm (measured using HOBO 0.2 mm tipping bucket rain gauge, measured as tips per hour). The incoming shortwave radiation reached 755 W m^-2^ (hourly average measured using a Zipp and Konen CNR4 Semi-Hemispheric Radiometer, logged with Campbell CR1000X), representing typical summer conditions in the ablation zone of the Greenland Ice Sheet (Fausto et al., 2021) (see Supplementary Data S1 for full weather station data).

### 2.2 Sample collection

On each sampling day, sediment from each cryoconite hole was collected in a 2 mL cryotube using a polycarbonate aquarium pipette and frozen at -80°C within an hour after sampling. At each of the five ice sites, the top 2 cm of surface ice was scraped off using an ice ax, selecting a similar, but pristine patch of ice around the site on each sampling day. The scraped ice was collected in Whirl-pak bags, melted in a 10°C water bath (as recommended by previous study by Peter et al., 2024), and 400 mL of each sample was filtered through Sartorius cellulose nitrate filters (0.2 μm). The filters with retained biomass were placed in cryotubes and frozen at -80°C until DNA/RNA coextraction. The average time between sampling and preservation of the ice samples was 6 h.

### 2.3 Coextraction of DNA and RNA

Samples (70 in total) from seven sampling days (July 28, August 1, 3, 7, 10, 13, 18) were used for coextraction of RNA and DNA using the NucleoBond RNA Soil Mini kit and the accompanying DNA set (#740142 and #740143, Macherey-Nagel, Germany). The entire ice filter or approximately 0.5 g of cryoconite sediment was used for extraction. Nucleotide extraction followed the manufacturer's instructions, supplemented with 100 μL of the proprietary ‘Buffer OPT' (Macherey-Nagel, Germany). Following the coextraction of DNA and RNA, concentrations were measured using a Qubit 4 fluorometer with the 1X dsDNA (HS) and the RNA (HS) assays respectively (Invitrogen, USA). The integrity and fragment size distribution of the extracted RNA were evaluated on a Tapestation 4,150 (Agilent Technologies, Denmark) using the RNA Screentapes and reagents. The extracted RNA was treated with the RapidOut DNA Removal kit (Thermo Scientific) following the manufacturer's instructions, to remove residual DNA before library preparation.

### 2.4 Total RNA sequencing

RNA libraries were prepared with the NEBNext Ultra II Directional RNA Library Prep with Sample Purification Beads (E7765, New England Biolabs, USA) following the manufacturer's instructions with these options: 7 min fragmentation time, 5-fold Adapter Dilution, seven cycles of final amplification. The libraries were run in a TapeStation 4150 with the D1000 Screentape and reagents to check for insert size distribution and presence of adapter-primer dimers. After verification, the libraries were pooled equimolarly and sequenced on a NextSeq 500 (Illumina) using the 300 cycles, v2.5 (151 bp pair-end sequencing) chemistry. The resulting fastq files were processed through our in-house automated total RNA analysis pipeline (https://zenodo.org/badge/latestdoi/546561474) as described in Scheel et al. (2023). Briefly, raw reads were quality-controlled with TrimGalore (https://www.bioinformatics.babraham.ac.uk/projects/trim_galore/), the SSU and mRNA reads were separated using SortMeRNA (Kopylova et al., 2012) and mRNA reads assembled using Trinity (Grabherr et al., 2011).

### 2.5 Nanopore metagenome sequencing

DNA extracts from August 1st were selected for metagenome sequencing based on their high RNA and DNA concentrations (Supplementary Data S2). The five cryoconite and five ice samples were pooled respectively and libraries were prepared using the Oxford Nanopore Technologies Ligation sequencing DNA V14 (SQK-LSK114) kit following the manufacturer's instructions. The two sample pools were sequenced on separate PromethION R10.4 flowcells, controlled by MinKNOW v.23.11.4. The resulting pod5 files were basecalled using Dorado v.0.4.3 (https://github.com/nanoporetech/dorado) with a minimym Q score of 12 (–min-qscore 12), and the dna_r10.4.1_e8.2_400bps_hac@v4.2.0model. Adapters were trimmed from the raw fastq files using Porechop v.0.2.4 (https://github.com/rrwick/Porechop) under default settings. The trimmed reads were used for metagenomic assemblies using Flye 2.9 (Kolmogorov et al., 2020) utilizing the options: –meta -i 5 –nano-hq. The metagenomic assemblies were partitioned with whokaryote v.1.1.2 (Pronk and Medema, 2022) to separate the eukaryotic and the prokaryotic contigs using the tiara-integrated model and the default minimum contig length of 5,000 bp.

### 2.6 MAG assembly and classification

The prokaryotic contigs were processed further with metaWRAP v.1.3.2 (Uritskiy et al., 2018) using the binners metaBat2 (Kang et al., 2019) and maxbin2 (Wu et al., 2016), and further depreplicating and combining for high quality (>90% completeness, < 5% contamination) metagenome-assembled genomes (MAGs) within metaWRAP (Uritskiy et al., 2018; Bowers et al., 2017). Taxonomic identification of all prokaryotic MAGs was done with GTDB-Tk v2.3.2 (Chaumeil et al., 2022).

### 2.7 Genome mining

AntiSMASH v. 6.1.1 (Blin et al., 2021) was used to detect BGCs. After sorting by Whokaryote, prokaryote and eukaryote contigs were treated separately, using prodigal (Hyatt et al., 2010) and glimmerhmm (Majoros et al., 2004) as gene finding tools, respectively. For eukaryote contigs, fungiSMASH was enabled with the flag “–taxon fungi.” The remaining unclassified contigs were run using both methods. AntiSMASH results were parsed using multiSMASH (Reitz, 2024). Similarity networks of the predicted BGCs were constructed using BiG-SCAPE version 1.1.5 (Navarro-Muñoz et al., 2020), including the MIBiG v.3.1 dataset (Terlouw et al., 2023). The “glocal” alignment mode was used. The default 0.30 and 0.70 cutoff values were used to create gene cluster families (GCFs) and gene cluster clans (GCCs). BiG-MAP (Pascal Andreu et al., 2021) was used to map the metatranscriptomic reads to the core regions of the identified BGCs, using default parameters. The coverage of the core genes of an expressed BGC was determined by subtracting the number of non-covered bases (ncb) from the total length of the cluster (cl): *Coverage* = (*cl*−*ncb*)/*cl*.

The online version of antiSMASH v.7 (Blin et al., 2023) and PRISM (Skinnider et al., 2020) were used to visualize and investigate the most expressed BGCs for cryoconite and ice. Cytoscape v.3.9.1 (Shannon et al., 2003) was used to visualize similarity networks based on BiG-SCAPE results. Clinker (Gilchrist and Chooi, 2021) was used to construct gene cluster comparison figures. R scripts used to create plots are provided in the Supplementary Data S3.

## 3 Results

### 3.1 Sequencing yield

The total RNA sequencing produced 692,276,652 reads from the ice surface samples that were then used for coassembly of all 35 samples taken. The mRNA reads were assembled into 105,903 contigs over 500 bp. The cryoconite samples produced 718,838,908 reads, and the mRNA reads were assembled into 77,276 contigs over 500 bp. The cryoconite metagenome assembly contained 1.8 gigabases, whereas the ice metagenome assembly amounted to 1.9 gigabases. After sorting, the ice surface metagenome contained 16,857 bacterial and 62,844 eukaryotic contigs, leaving 1,218 contigs unclassified. The cryoconite metagenome included 50,132 bacterial and 13,641 eukaryotic contigs, and 1,032 unclassified (Supplementary Data S4).

### 3.2 Observed biosynthetic potential

Mining both metagenomes yielded 1,828 predicted biosynthetic gene clusters (BGCs). One nonribosomal peptide synthetase (NRPS) BGC was identified in an unclassified contig from the cryoconite metagenome, but since it was identified after annotation with prodigal, it was treated as a BGC of prokaryote origin. The cryoconite metagenome yielded 1,384 BGCs, of which 1,361 were from prokaryote contigs and 23 were from eukaryote contigs. The ice metagenome yielded 444 BGCs, of which 384 were of prokaryote origin and 60 were from eukaryote contigs. Of the predicted gene clusters, 53% are likely complete, as they were not located at the edge of a contig (Supplementary Data S4).

Out of the 71 cluster types detectable by antiSMASH, 46 were found in the collection of BGCs (Supplementary Data S4). The most notable difference in the distribution of compound classes, as determined by BiG-SCAPE, is found for eukaryotes between ice and cryoconite. The distribution of prokaryote BGCs by class is more similar between environments (Supplementary Figure S5).

### 3.3 Similarity network analysis of biosynthetic diversity

**Figure 2:**
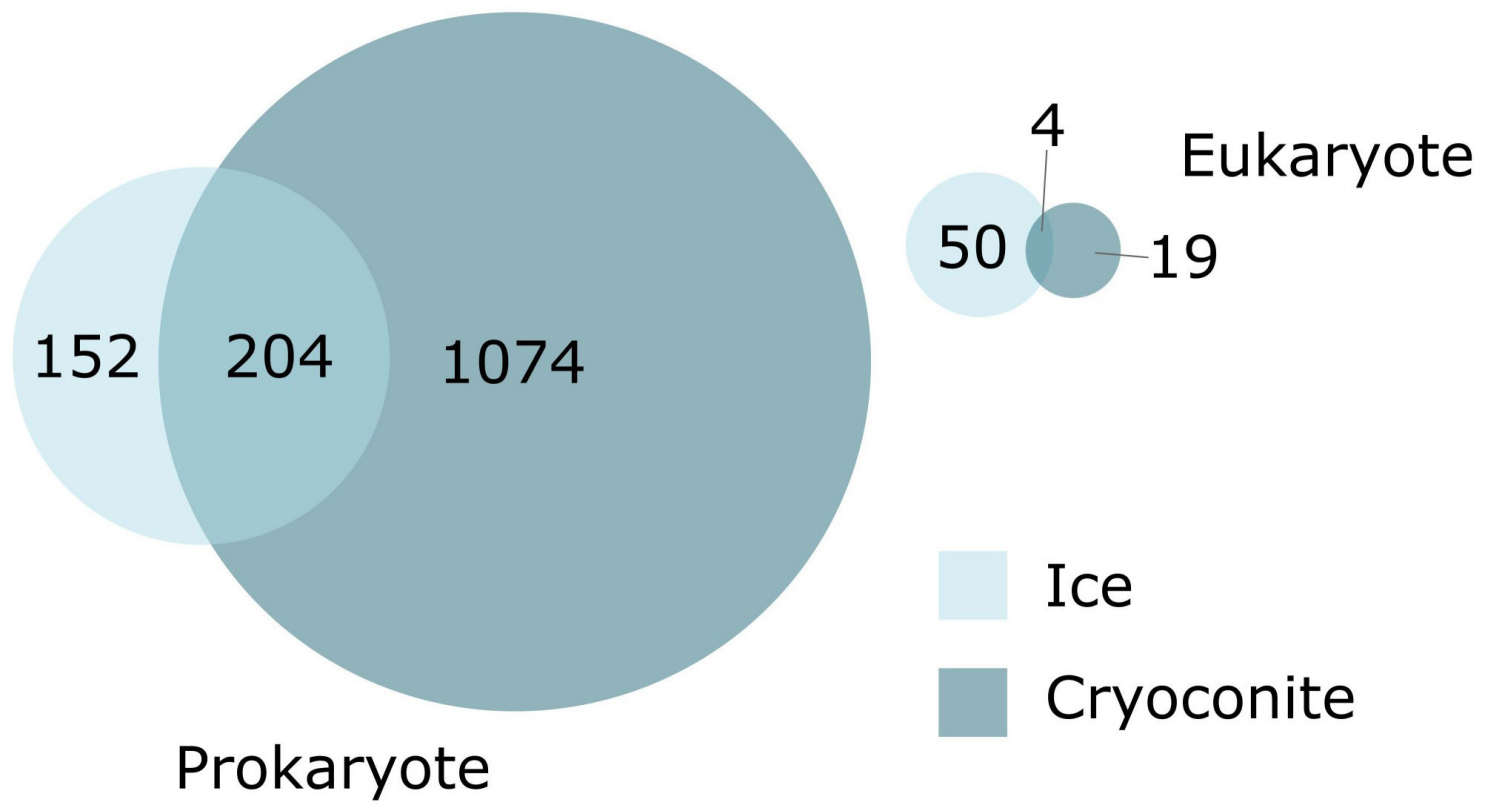
Distribution and overlap of gene cluster families (GCFs) by environment.

The BGCs were grouped into 1,711 Gene Cluster Families (GCFs) based on their structural similarity (Supplementary Figure S6). The BGCs from the cryoconite and ice metagenome formed 1,301 and 410 GCFs, respectively. Of these GCFs, 1,258 contain singleton BGCs. There were fewer shared eukaryotic GCFs between the two environments; four GCFs were found in both. For prokaryote GCFs, 204 were found in both the ice and cryoconite metagenomes (Figure 2). While the MIBiG dataset (Terlouw et al., 2023) was included in the BiG-SCAPE run, no MIBiG BGC was found to cluster in a GCF with the ice and cryoconite BGCs.

### 3.4 Expression of BGCs

Using BiG-MAP, 1,453 non-redundant representative BGCs were selected for the mapping process out of the families assigned by BiG-SCAPE. Of these selected representative BGCs, 59% had non-zero expression, meaning that its core genes had expression of over 0 reads per kilobase per million mapped reads (RPKM) in at least one of the samples. Of the non-redundant set of BGCs, 1,384 originated from prokaryote contigs, and 59% of those were expressed in at least one of the samples. Similarly, out of the 69 representative BGCs from eukaryote contigs, 62% showed expression in at least one sample throughout the melting season. In cryoconite, 84 BGCs were expressed in at least half of the samples, compared to only 9 BGCs in ice (Supplementary Data S4).

Of the observations of expressed BGCs with non-zero expression, the overall average RPKM across sample days and sites was 554, with a median of 147. When expressed, gene clusters originating from prokaryote contigs gave a smaller average RPKM (364) than those originating from the eukaryotic contigs (2,724) (Supplementary Data S4).

**Figure 3:**
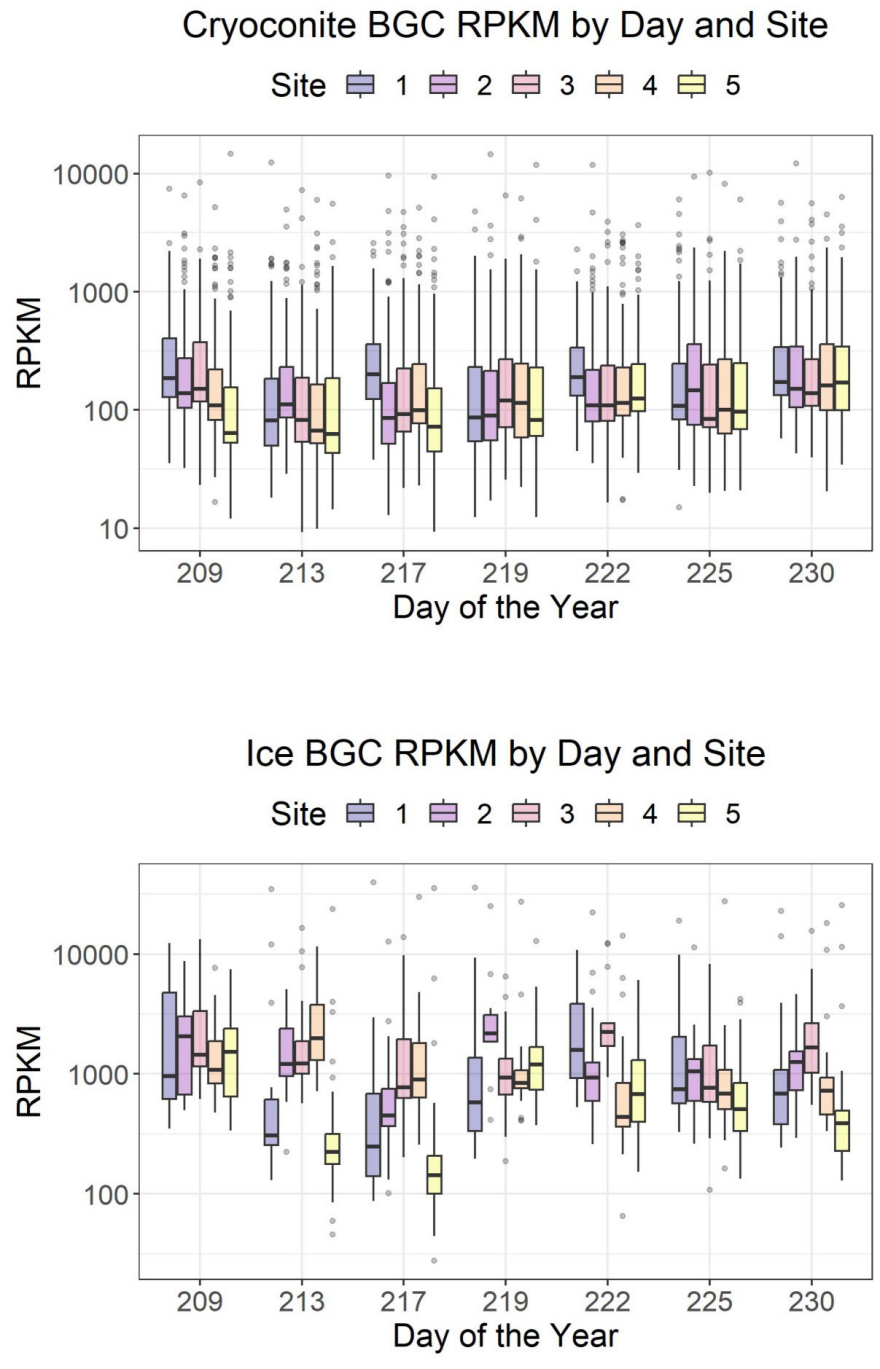
Distribution of reads per kilobase per million mapped reads (RPKM) of core genes of expressed BGCs over time.

Expressed BGCs in ice surface samples had an average RPKM of 719, while BGCs in cryoconite had an average RPKM of 120. The variation between sites is larger for the ice than for the cryoconite, and the mean expression during the sample period remained relatively stable, especially for the cryoconite samples (Figure 3). Temporal and site-specific patterns were not evident in an NMDS made with those gene clusters that were expressed in over half of the samples (Supplementary Figure S7).

### 3.5 Overview of the most highly expressed BGCs

**Figure 4:**
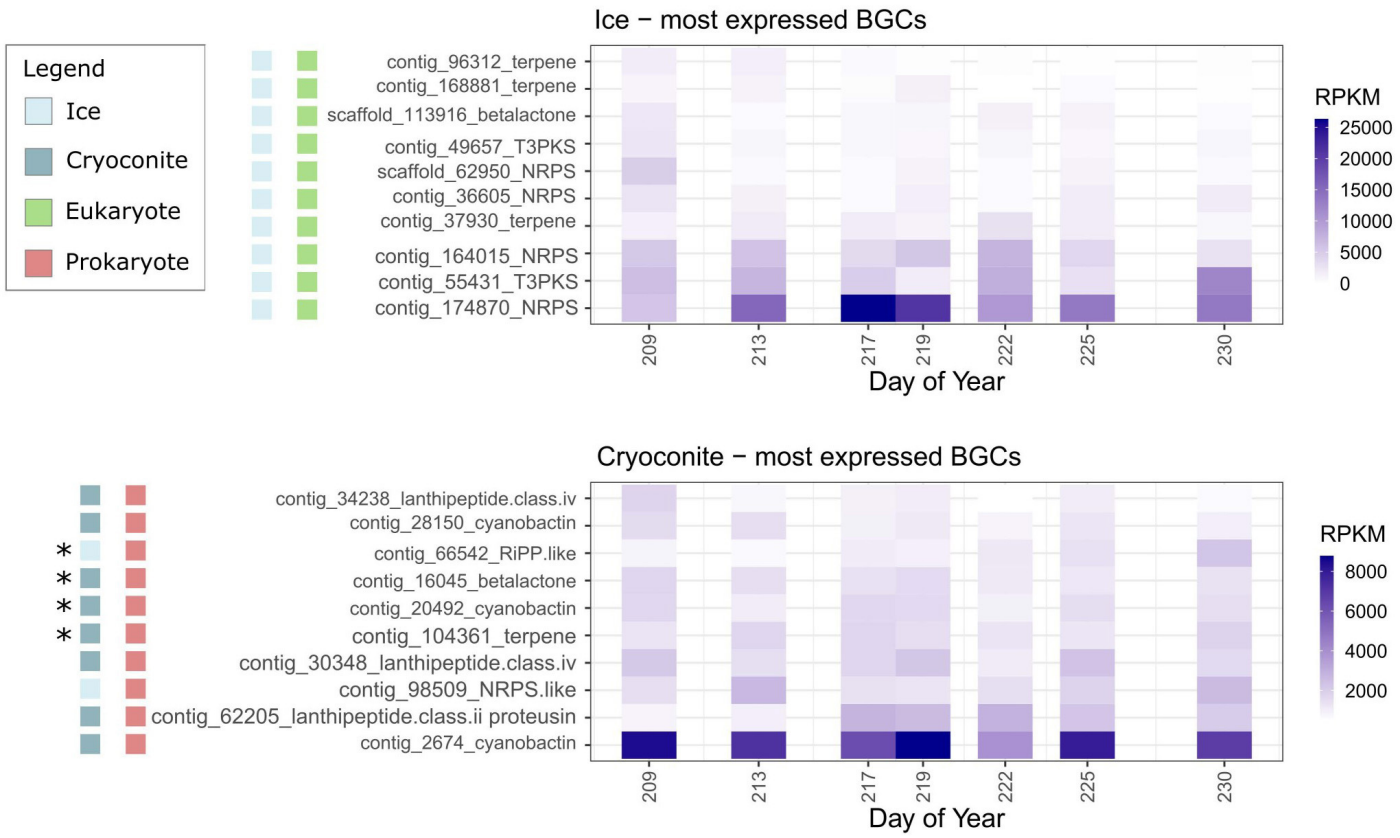
Average expression of core genes (RPKM) of the ten most expressed BGCs in ice and cryoconite for each sample day. Asterisks indicate BGCs also located in MAGs (Supplementary Figure S11).

Based on RPKM values, the 10 most expressed BGCs in cryoconite samples were of prokaryotic origin, whereas the 10 most expressed BGCs in ice samples were of eukaryotic origin (Figure 4). On the sample days where expression was observed, the coverage of the core genes of the 10 most expressed gene clusters for ice and cryoconite was on average 18% and 45%, respectively. Complete coverage of the core genes was only achieved for two BGCs, both in cryoconite samples (Supplementary Data S4).

**Figure 5:**
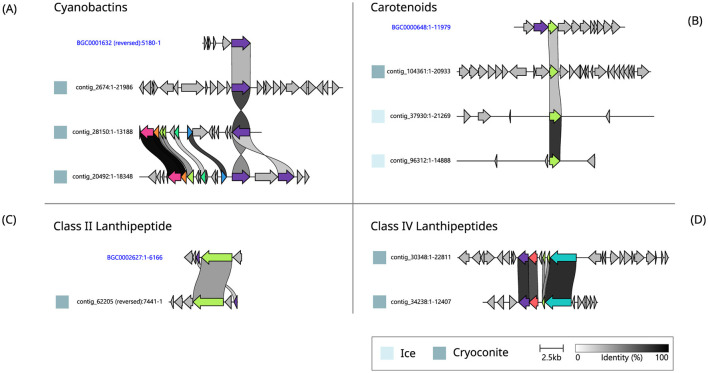
Pairwise alignment of homologous genes found in BGCs from cryoconite and ice and reference BGCs from the MiBIG repository (names in blue). The shown BGCs belong to the Cyanobactins **(A)**, Carotenoids **(B)**, Class II **(C)** and IV **(D)** Lanthipeptides.

Three of the most expressed BGCs (based on RPKM values) in cryoconite samples, including the highest expressed BGC, were cyanobactins. These BGCs have similarities to a BGC from the MiBIG database (ref. BGC0001632) (Figure 5A). The second most expressed BGC in cryoconite, located on contig 62,205, contains two genes that have similarity to MiBIG gene cluster BGC0002627 (Figure 5C). There were two additional lanthipeptide BGCs, with comparable gene cluster organization (Figure 5D) but no significant similarity to MiBIG reference BGCs. Another, NRPS-like, BGC from the ice metagenome was the third most expressed BGC in cryoconite, but had no significant matches to MiBIG BGCs. Lastly, a betalactone BGC was among the most expressed, with one gene similar to *fdrC* from a *Streptomyces* BGC (ref. BGC0001928).

Three of the most expressed BGCs in ice, and one in cryoconite, encode carotenoids. Three of them align with one gene in the MiBIG reference BGC0000648 (Figure 5B). The most expressed and third most expressed BGCs in ice are NRPSs with no significant matches to MiBIG BGCs. Two additional NRPS type BGCs include a single NRPS gene which showed similarity to a BGC encoding choline production (BGC0002276). Secondly, a single NRPS was found on contig 36,605, with no significant matches to MiBIG BGCs. Additional BGCs without significant matches to the database were a single betalactone and a type III polyketide synthase.

### 3.6 Presence of expressed BGCs in MAGs

A total of 96 high-quality bacterial MAGs were assembled. The majority (79) were derived from the cryoconite metagenome, and 17 originated from the ice metagenome. Pseudomonadota was the dominant phylum in both the cryoconite and ice MAGs out of 12 and 10 different phyla, respectively (Supplementary Data S4).

We investigated whether the most expressed prokaryotic BGCs were present within the assembled MAGs. Of the top 10 most expressed BGCs in cryoconite, four were found present in a MAG. Two of these, a betalactone and terpene BGC, were present in a *Phormidesmis* MAG from the ice metagenome. The other two, encoding a RiPP-like and cyanobactin product, were found in MAGs from the cryoconite metagenome, from the family Acetobacteraceae and Nostocaceae, respectively (Supplementary Figure S8).

## 4 Discussion

In this study, we mined metagenomes from ice and cryoconite to identify encoded biosynthetic potential, and compared them to metatranscriptomes to validate the expression of this biosynthetic potential by microbes in the melting surface ice and cryoconite holes of the Greenland ice sheet. We created an inventory of 1,453 biosynthetic gene clusters, and 59% of them were observed in the metatranscriptomics data, indicating that a substantial portion of the encoded biosynthetic machinery was expressed during the melting season. The benefits of long-read sequencing technologies for genome mining have been demonstrated before (Huang et al., 2023; Van Goethem et al., 2021; Waschulin et al., 2022; Sánchez-Navarro et al., 2022). Still, many regions, including some of the most expressed ones, were located on a contig edge. We have tried to mitigate this limitation by focusing on the expression of relevant core genes of each BGC. We demonstrate the benefit of sorting contigs into prokaryote and eukaryote datasets, as proposed by Pronk and Medema (2022). This enabled us to specifically apply fungiSMASH to accurately predicted eukaryote sequences, rather than assuming the entire metagenome to be bacterial and potentially missing, or misidentifying eukaryotic gene clusters. We found eukaryotic BGCs to be the most expressed in ice, not surprisingly, considering that the ice is dominated by eukaryotic members (Jaarsma et al., 2023a), and they therefore are likely playing a bigger role in the ice community ecology.

### 4.1 Greenland Ice Sheet microbes use their biosynthetic potential *in situ*

In this study, we confirm that the ice metagenome contained mainly eukaryotic DNA, and the cryoconite metagenome was dominated by prokaryotic DNA. This corresponds to previous findings of the importance of eukaryotes on the ice surface and of prokaryotes in cryoconite holes of the Greenland Ice Sheet (Jaarsma et al., 2023a). However, the majority of encoded BGCs were of prokaryotic origin in both environments. There was also a large overlap in gene cluster families (GCFs) of prokaryote origin found in ice and cryoconite, suggesting that this biosynthetic potential is found in bacteria that are abundant in both types of supraglacial environments. This finding would match the previous observations of MAGs that were not bound to a specific supraglacial environment, but omnipresent (Jaarsma et al., 2023a). We expect eukaryotic BGCs to be underrepresented in our study as fungiSMASH is designed in the first place to detect fungal BGCs, while our ice samples were dominated by glacier ice algae (in prep). Despite the large representation of prokaryotic BCGs, all 10 of the most expressed BGCs in ice are of eukaryotic origin, again reflecting the important role of eukaryotes on the ice surface, not just in abundance, but also in activity. On average, much higher expression levels were observed for eukaryotic BGCs compared to prokaryotic BGCs, though this difference was caused by a small selection of BGCs that were highly expressed in ice.

While the total expression levels appeared to be relatively stable during the sampling period, the ice exhibits larger spatial variation as a result of the destructive nature of the sampling of ice. Where the same cryoconite holes could be re-sampled throughout the sampling period, each sampled ice surface is unique. Consequently, the interpretation of potential temporal variability is obscured by these substantial spatial variations. We also acknowledge that the slower sample processing of ice due to the inevitable melting step is not ideal for transcriptomic sequencing. Yet we have adhered to the most optimal method for melting ice with the least disturbance (Peter et al., 2024). More dramatic differences in community composition occur over the different seasons of the year (Winkel et al., 2022), and therefore, we would expect that the expression of BGCs during the winter season, as well as the early and late phases of the melt season, would be different from that during the peak melting season. Additional studies, outside of the typical fieldwork season, are required to test this hypothesis.

We have previously mapped the biosynthetic potential in bacterial MAGs and isolate genomes from the Greenland Ice Sheet (Jaarsma et al., 2023b) but did not consider whether biosynthetic potential is actively expressed *in situ*. Previous studies have investigated a range of metabolites related to cell defense and communication produced on the ice surface and in cryoconite habitats (Doting et al., 2022, 2024; Gokul et al., 2023), and our study adds to the evidence for the production of natural products on the Greenland Ice Sheet. Almost 60% of the total biosynthetic potential encoded in the metagenomes was expressed in at least one of the samples, and this share of non-silent BGCs was very similar for those of eukaryote and prokaryote origin. A substantial portion of the biosynthetic potential is being used by the organisms, which is in contrast to findings under laboratory conditions, where many BGCs are silent (Rutledge and Challis, 2015). Previous studies into *in situ* expression of BGCs have found similar results. For example, a study found that about 30% of the secondary metabolic genes in their dataset was expressed in samples from the Yellow Sea, China (Huang et al., 2023). Another study reports constitutive expression of 6% of secondary metabolic genes in their dataset during lab-simulated rain events in intact desert biocrust samples from Moab, Utah (Van Goethem et al., 2021).

While the screening hypothesis allows for an explanation in which not all BGCs have a biological function (Firn and Jones, 2003), some of the BGCs identified in this study were continuously expressed throughout the sampling period, suggesting an important function in the ecosystem. By focusing on the most highly expressed BGCs, we aimed to cover those that are most likely to have biological relevance. Currently, there are many unknowns surrounding the influence of microbial interactions on glacier ice algae responsible for the biological darkening of the Greenland Ice Sheet (Halbach et al., 2023). Understanding the ecological roles of these BGCs is crucial, as they may mediate interactions between the organisms that produce them and the dominant pigmented glacier ice algae. However, predicting the exact natural product encoded in each gene cluster, including the most expressed ones, remains a challenge. In some cases, we did find clues toward the producing organisms. For instance, there was abundant expression of cyanobactins in cryoconite, and one of these BGCs was found in a Nostocaceae MAG. The similarity of these cyanobactin BGCs to the reference BGC, which encodes kawaguchipeptin A, produced by the cyanobacterium *Microcystis aeruginosa* (Parajuli et al., 2016), is limited to one gene, encoding an S8 peptidase (Figure 5A). The gene cluster found on contig 20,492 contains two copies of this peptidase gene, and shares five additional homologous genes with the cluster found on contig 28,150, together likely encoding a complete RiPP biosynthesis pathway.

The second most expressed BGC in cryoconite showed similarity to a BGC that encodes kamptornamide, a class II lanthipeptide produced by the cyanobacterium *Kamptonema sp*. PCC 6506 (Figure 5C). PRISM (Skinnider et al., 2020) also identified this gene cluster from cryoconite to encode a class II lanthipeptide. The cluster includes a LanM-like enzyme, typically responsible for dehydration and cyclization steps that are part of lanthipeptide maturation (Willey and Van Der Donk, 2007). PRISM flags the other gene as a proteusin precursor peptide that this enzyme could modify. After running BLASTp on both gene products, 76–78% similarity was found to sequences from *Phormidesmis priestleyi* (accession no. PZO42957.1 and PZO50565.1), similarly annotated as an NHLP leader peptide family natural product precursor and a type 2 lanthipeptide synthetase LanM.

Additionally, the betalactone BGC that was among the highest expressed BGCs in cryoconite showed similarity to a BGC encoding a fluorometabolite (Ma et al., 2015), which are a rare type of natural products (Deng et al., 2004). After running BLASTp, the sequence showed 99% similarity to oxidoreductases from Leptolyngbyaceae (MBC7825090.1) and *Phormidesmis priestleyi* (WP_068815775.1), and indeed this BGC was present in a *Phormidesmis* MAG from the ice metagenome. Similarly, a phytoene synthase that is part of the carotenoid BGC expressed in cryoconite was identical to that of *P. priestleyi* (WP_068815503.1). This cyanobacterium dominates polar cryoconite (Murakami et al., 2022) and is considered an ecosystem engineer (Gokul et al., 2019). In an accompanying study also using this dataset, we have found cyanobacteria to dominate the active community in nearly all cryoconite samples throughout the sampling period (in prep).

The carotenoid biosynthesis machinery that is highly expressed in ice shows 70–80% similarity to proteins of various taxa of plants and algae, suggesting that it might be encoded by Streptophyta glacier ice algae, and/or by chlorophyte snow algae. Therefore, it appears that some of these highly expressed BGCs might be encoded by key organisms of the supraglacial habitats. Indeed, we have found Streptophyta and Chlorophyta to be consistently dominant in the active community in the ice samples throughout this seasonal study (in prep). In addition, we previously found carotenoid BGCs encoded in (meta)genomes from Greenland Ice Sheet microorganisms and hypothesized that supraglacial microbes may use carotenoid pigments to shield themselves from the harsh UV radiation encountered in this environment (Jaarsma et al., 2023b). Furthermore, carotenoids also play a role in the regulation of membrane fluidity in low temperatures (De Maayer et al., 2014). In this study, we identified one particular gene that was shared among the highly expressed carotenoid BGCs in ice and cryoconite, encoding a phytoene synthase, which is a key enzyme for carotenoid biosynthesis (Zhou et al., 2022). Phytoene is an intermediate in carotenoid biosynthesis, but itself also absorbs UV light (Armstrong, 1999), a possible indication of a role in UV protection, which would be a relevant environmental adaptation for the Greenland Ice Sheet. Phytoene synthases were also abundant in the biosynthetic potential of Antarctic soil bacteria (Waschulin et al., 2022), and also in warmer environments (Huang et al., 2023; Van Goethem et al., 2021).

It is also notable to observe biosynthetic machinery involved in the production of modified peptides, in particular non-ribosomal peptide synthetases and ribosomally synthesized, post-translationally modified peptide (RiPP) modification enzymes, among the most expressed BGCs in both supraglacial environments. In addition to the RiPP BGCs mentioned above, among the most expressed in cryoconite was a BGC from contig 66,542 from the ice metagenome, also found in an Acetobacteraceae MAG from the cryoconite metagenome. This BGC encodes a radical S-adenosyl-l-methionine (SAM) C-methyltransferase (TIGRFam: TIGR03975.1), which are typically involved in RiPP maturation (Morinaka et al., 2014). Additionally, two lanthipeptide BGCs contained a Class IV lanthipeptide modification enzyme. An NRPS gene found to be highly expressed in ice showed similarity to a L-2-aminoadipate reductase from *Phenoliferia* (Genbank ref. KAK4703385.1), suggesting that it might be an NRPS-like carboxylic acid reductase (CAR) (Hai et al., 2019).

The unusual modifications found in NRPs and RiPPs, including cyanobactins, confer them with remarkable structural diversity, and as a result, these modified peptides have a wide range of observed biological activities in lab assays (Schwarzer et al., 2003; Arnison et al., 2013). Less is known about actual ecological functions, but several roles of RiPPs in the community are documented, including competition and defense, quorum sensing, biofilm formation, and metal scavenging (Li and Rebuffat, 2020), all of which could provide an adaptive advantage in a harsh oligotrophic habitat such as the ice sheet surface. Similarly, NRPs are involved in various ecological interactions, including competitive behaviors and iron chelation (Schwarzer et al., 2003). It remains unclear if the peptide products of the BGCs expressed in our study are mainly used in competition, or if these modified peptides have additional functions for environmental adaptation.

### 4.2 The bioprospecting potential of Greenland Ice Sheet microbes

The bulk of BGCs identified in this study are of unknown function, lacking similarity to previously described biosynthesis machinery in the MiBIG database. This illustrates the untapped biosynthetic potential harbored by an underexplored extreme environment such as the Greenland Ice Sheet. There is potential for further investigation of many of these BGCs, with the possibility of testing their products for activity, for example, through heterologous expression (Kadjo and Eustáquio, 2023). The components of this biosynthetic machinery, especially those involved in the synthesis of RiPPs, NRPs, and polyketides, are particularly well-suited for combinatorial biosynthesis due to their modular nature, offering opportunities for producing novel chemical diversity (Baltz, 2018; Sardar and Schmidt, 2016; Fischbach and Walsh, 2006). Examples of such useful biosynthesis machinery include tailoring enzymes like radical SAM (Fu and Balskus, 2020) and carboxylic acid reductase (CAR) (Finnigan et al., 2017) found in some of the highest expressed gene clusters in this study.

To our knowledge, this is the first study to investigate the expression of biosynthetic gene clusters in a supraglacial microbial habitat. We demonstrate that microbes found on the surface of the Greenland Ice Sheet not only possess diverse biosynthetic potential, but also actively express a substantial portion of it. Some of the most expressed BGCs seem to be produced by key ecosystem engineers in these environments, illustrating the potential ecological importance of these thus far unknown biosynthetic gene clusters. The discovery of numerous unknown BGCs in these supraglacial habitats suggests interesting new avenues for research to enhance both our understanding of its role in microbial ecology and its biotechnological potential.

## Data Availability

The original contributions presented in the study are publicly available. This data can be found at: https://www.ncbi.nlm.nih.gov, accession number PRJNA1160058.
