# Supplementary material for: The encoded and expressed biosynthetic potential of Greenland Ice Sheet microbes
Source: Front Microbiol. 2025 Jul 31;16:1620548. doi: 10.3389/fmicb.2025.1620548 (PMC12350317; doi:10.3389/fmicb.2025.1620548)
Supplement: Supplementary file 1 [file Data_Sheet_1.pdf]

We used R version 4.1.2 (R Core Team 2021) and the following R packages: ggsci v. 3.0.0 (Xiao 2023), grateful v. 0.1.11 (Rodríguez-Sánchez, Jackson, and Hutchins 2022), hrbrthemes v. 0.8.0 (Rudis 2020), microbiome v. 1.16.0 (Lahti and Shetty 2012-2019), phyloseq v. 1.38.0 (McMurdie and Holmes 2013), reshape2 v. 1.4.4 (Wickham 2007), rwantshue v. 0.0.3 (Hösler" 2023), scales v. 1.2.1 (Wickham and Seidel 2022), tidyverse v. 1.3.2 (Wickham et al. 2019), vegan v. 2.6.4 (Oksanen et al. 2022), VennDiagram v. 1.7.3 (Chen 2022), viridis v. 0.6.2 (Garnier et al. 2021).

Package citations

Chen, Hanbo. 2022. VennDiagram: Generate High-Resolution Venn and Euler Plots. https://CRAN.R-project.org/package=VennDiagram.

Garnier, Simon, Ross, Noam, Rudis, Robert, Camargo, et al. 2021. viridis - Colorblind-Friendly Color Maps for r. https://doi.org/10.5281/zenodo.4679424.

Hösler", "Christoph. 2023. Rwantshue: R Adaptor for the "i Want Hue" Color Palette Generator.

Lahti, Leo, and Sudarshan Shetty. 2012-2019. "Microbiome r Package."

McMurdie, Paul J., and Susan Holmes. 2013. "Phyloseq: An r Package for Reproducible Interactive Analysis and Graphics of Microbiome Census Data." PLoS ONE 8 (4): e61217. http://dx.plos.org/10.1371/journal.pone.0061217.

Oksanen, Jari, Gavin L. Simpson, F. Guillaume Blanchet, Roeland Kindt, Pierre Legendre, Peter R. Minchin, R. B. O'Hara, et al. 2022. Vegan: Community Ecology Package. https://CRAN.R-project.org/package=vegan.

R Core Team. 2021. R: A Language and Environment for Statistical Computing. Vienna, Austria: R Foundation for Statistical Computing. https://www.R-project.org/.

Rodríguez-Sánchez, Francisco, Connor P. Jackson, and Shaurita D. Hutchins. 2022. Grateful: Facilitate Citation of r Packages. https://github.com/Pakillo/grateful.

Rudis, Bob. 2020. Hrbrthemes: Additional Themes, Theme Components and Utilities for 'Ggplot2'. https://CRAN.R-project.org/package=hrbrthemes.

Wickham, Hadley. 2007. "Reshaping Data with the reshape Package." Journal of Statistical Software 21 (12): 1-20. http://www.jstatsoft.org/v21/i12/.

Wickham, Hadley, Mara Averick, Jennifer Bryan, Winston Chang, Lucy D'Agostino McGowan, Romain François, Garrett Grolemund, et al. 2019. "Welcome to the tidyverse." Journal of Open Source Software 4 (43): 1686. https://doi.org/10.21105/joss.01686.

Wickham, Hadley, and Dana Seidel. 2022. Scales: Scale Functions for Visualization. https://CRAN.R-project.org/package=scales.

Xiao, Nan. 2023. Ggsci: Scientific Journal and Sci-Fi Themed Color Palettes for 'Ggplot2'. https://CRAN.R-project.org/package=ggsci.

```
# Load the necessary libraries

library(ggplot2)

library(dplyr)

library(reshape2)

library(viridis)

library(scales)



# Load the data from the Excel file

data <- read.csv("bigmap.csv")



# Step 1: Split the dataset by environment (Ice and Cryoconite)

ice_data <- data %>% filter(ENV == "ice")

cryoconite_data <- data %>% filter(ENV == "cryoconite")


# Function to select the top N gb columns

select_top_n_gb_columns <- function(df, top_n) {

  # Select all gb columns
```

```
  selected_columns <- grep("^gb", colnames(df))

  # Select the top N columns based on the sum

  top_n_columns <- names(sort(colSums(df[, selected_columns]), decreasing =
TRUE)[1:top_n])

  return(df[, c("DAYOFYEAR", "SITE", top_n_columns)])

}


# Step 2: Select the top 10 gb columns for both Ice and Cryoconite

top_n <- 10

ice_top_n_gb <- select_top_n_gb_columns(ice_data, top_n)

cryoconite_top_n_gb <- select_top_n_gb_columns(cryoconite_data, top_n)


# Step 3: Average values by sample site and day of the year

ice_averaged_data <- ice_top_n_gb %>%

  group_by(DAYOFYEAR) %>%

  summarise(across(starts_with("gb"), mean))


cryoconite_averaged_data <- cryoconite_top_n_gb %>%

  group_by(DAYOFYEAR) %>%

  summarise(across(starts_with("gb"), mean))



# Create a custom color palette

colors <- c("white", "darkblue")
```

```
# Function to create heatmap

create_heatmap <- function(data, title, legend_title) {

  data_melted <- melt(data, id.vars = "DAYOFYEAR")

  ggplot(data_melted, aes(x = DAYOFYEAR, y = variable, fill = value)) +

    geom_tile() +

    scale_fill_gradientn(colors = colors, na.value = "white", name = legend_title) + # Use the
custom colors

    labs(title = title, x = "Day of Year", y = "") +  # Updated to top 10 as per your script

    theme_bw() +  # Change theme to black and white

    theme(plot.background = element_rect(fill = "white"),

          text = element_text(size = 40),  # General text size for the plot

          axis.title.x = element_text(size = 40),  # X axis title size

          axis.title.y = element_text(size = 40),  # Y axis title size

          axis.text.x = element_text(size = 40, angle = 90, vjust = 0.5, hjust=1),  # X axis text size

          axis.text.y = element_text(size = 40),  # Y axis text size

          legend.title = element_text(size = 40),  # Legend title size

          legend.text = element_text(size = 36)) +  # Legend text size

    guides(fill = guide_colourbar(barwidth = 2, barheight = 10)) +  # Adjust the size of the color
bar

    scale_x_continuous(breaks = unique(data_melted$DAYOFYEAR))  # Set x-axis labels for
each day

}




# Create heatmaps
```

```
ice_heatmap <- create_heatmap(ice_averaged_data, "Ice - most expressed BGCs", "RPKM")

cryoconite_heatmap <- create_heatmap(cryoconite_averaged_data, "Cryoconite - most expressed BGCs", "RPKM")


# Save the heatmaps as PNG files

ggsave(filename = "ice_heatmap.png", plot = ice_heatmap, width = 45, height = 20)

ggsave(filename = "cryoconite_heatmap.png", plot = cryoconite_heatmap, width = 45, height = 20)




# Save the heatmaps as PDF files

ggsave(filename = "ice_heatmap.png", plot = ice_heatmap, width = 45, height = 20)

ggsave(filename = "cryoconite_heatmap.png", plot = cryoconite_heatmap, width = 45, height = 20)




######################################################################

######################################################################


# BAR PLOTS and VENN


######################################################################
```

```
########################################################################

# Libraries
library(ggplot2)
library(dplyr)
library(hrbrthemes)
library(viridis)
library(forcats)
library(ggsci)

# bar plots
## load data

domclass <- read.csv("domclass.csv")
prokclass <- read.csv("prokclass.csv")
eukclass <- read.csv("eukclass.csv")

## Bar plot of classes by domain
ggsave("domclass.png", bg = 'white', width = 10, height = 7, print(
  domclass %>%
    ggplot(aes(fill=Class, y=Value, x=Origin)) +
    geom_bar(position="fill", stat="identity", lwd = 0, color = "white") +
scale_fill_frontiers(alpha = 0.6) +
```

```
  ggtitle("Distribution of BGC classes") +   theme_ipsum() +   xlab("") + ylab("") + coord_flip()
+

  theme(panel.grid.major = element_blank(),

        panel.grid.minor = element_blank(),

        plot.title = element_text(size=20),

        axis.text.y = element_text(size=15),

        legend.text = element_text(size = 15),

        legend.title = element_blank())))
```

```
## Bar plot of classes prok only

ggsave("prokclass.png", bg = 'white', width = 10, height = 7, print(

  prokclass %>%

  ggplot(aes(fill=Class, y=Value, x=Origin)) +

  geom_bar(position="fill", stat="identity", lwd = 0, color = "white") +
scale_fill_frontiers(alpha = 0.6) +

  ggtitle("Distribution of BGC classes (Prokaryotes)") +   theme_ipsum() +   xlab("") + ylab("")
+ coord_flip() +

  theme(panel.grid.major = element_blank(),

        panel.grid.minor = element_blank(),

        plot.title = element_text(size=20),

        axis.text.y = element_text(size=15),

        legend.text = element_text(size = 15),

        legend.title = element_blank())))
```

```
## Bar plot of classes euk only
```

```
ggsave("eukclass.png", bg = 'white', width = 10, height = 7, print(
  eukclass %>%
    ggplot(aes(fill=Class, y=Value, x=Origin)) +
    geom_bar(position="fill", stat="identity", lwd = 0, color = "white") +
scale_fill_frontiers(alpha = 0.6) +
    ggtitle("Distribution of BGC classes (Eukaryotes)") +   theme_ipsum() +   xlab("") + ylab("")
+ coord_flip() +
    theme(panel.grid.major = element_blank(),
          panel.grid.minor = element_blank(),
          plot.title = element_text(size=20),
          axis.text.y = element_text(size=15),
          legend.text = element_text(size = 15),
          legend.title = element_blank())))

############# VENN

library(VennDiagram)

x <- read.csv("prokvenn.csv")

lst1=list()
```

```
for(i in 1:ncol(x)) {
  lst1[[i]] <- x[ , i]
}

names(lst1)=colnames(x)
print(lst1)

venn.diagram(lst1, na = "remove", "prokvenn.png")



y <- read.csv("eukvenn.csv")

lst2=list()

for(i in 1:ncol(y)) {
  lst2[[i]] <- y[ , i]
}

names(lst2)=colnames(y)
print(lst2)

venn.diagram(lst2, na = "remove", "eukvenn.png")

########################################################################
########################################################################
```

```
# BOX plots

##################################################################
##################################################################



library(ggplot2)
library(dplyr)

# Load your data
data <- read.csv('bigmap.csv')

# Filter out zero-expression gene clusters and transform data to long format
data_long <- reshape2::melt(data, id.vars = c('run.ID', 'ENV', 'DAYOFYEAR', 'SITE'),
                    variable.name = 'Gene_Cluster', value.name = 'Expression')
data_long <- subset(data_long, Expression > 0)

# Split the data by environment
cryoconite_data <- subset(data_long, ENV == 'cryoconite')
ice_data <- subset(data_long, ENV == 'ice')



# Updated function to create boxplot with jitter and slightly reduced text size
create_boxplot_with_jitter <- function(data, title, file_name) {
  p <- ggplot(data, aes(x = factor(DAYOFYEAR), y = Expression, fill = factor(SITE))) +
    geom_boxplot(alpha = 0.3, outlier.size = 1) +  # Transparent boxplots, outliers visible
```

```
  scale_y_log10() +
  labs(title = title, x = "Day of the Year", y = "RPKM", fill = "Site") +
  scale_fill_viridis(discrete = TRUE, option = "C") +  # Colorblind-friendly palette
  theme_bw() +
  theme(
   text = element_text(size = 18),  # Slightly smaller base text size for readability
   axis.title = element_text(size = 16),  # Adjusted axis titles
   axis.text = element_text(size = 14),  # Adjusted axis labels
   legend.title = element_text(size = 16),  # Adjusted legend title
   legend.text = element_text(size = 14),  # Adjusted legend text
   plot.title = element_text(size = 18, hjust = 0.5),  # Slightly smaller plot title, centered
   plot.margin = unit(c(1, 1, 1, 1), "cm")  # Margins for proper spacing
  ) +
  theme(legend.position = "top")  # Legend on top for space efficiency
 print(p)  # Explicitly print the plot
}

# Create and save the plots
png("cryoconite_boxplot_fixed.png", width = 2000, height = 1500, res = 300)  # Larger size for better plot space
create_boxplot_with_jitter(cryoconite_data, "Cryoconite BGC RPKM by Day and Site", "cryoconite_boxplot_fixed.png")
dev.off()

png("ice_boxplot_fixed.png", width = 2000, height = 1500, res = 300)  # Larger size for better plot space
```

```
create_boxplot_with_jitter(ice_data, "Ice BGC RPKM by Day and Site",
"ice_boxplot_fixed.png")

dev.off()




######################################################################

######################################################################


# Expression stats


######################################################################

######################################################################



# Load necessary libraries

library(dplyr)


# Read the data from the CSV file

data <- read.csv("bigmap.csv")


# Function to calculate expression statistics

calculate_expression_stats <- function(df, name) {

  # Select gene columns (starting with "gb")
```

```r
gene_columns <- df %>% select(starts_with("gb"))

# Calculate the total number of gene clusters
total_genes <- ncol(gene_columns)

# Calculate the number of expressed genes (non-zero in at least one sample)
expressed_genes <- sum(colSums(gene_columns > 0) > 0)

# Calculate the number of never expressed genes
never_expressed_genes <- total_genes - expressed_genes

# Calculate the percentage of expressed and never expressed genes
percent_expressed <- (expressed_genes / total_genes) * 100
percent_never_expressed <- (never_expressed_genes / total_genes) * 100

# Extract only the values of expressed genes
expressed_values <- gene_columns[gene_columns > 0]

# Calculate the overall average RPKM for expressed genes
overall_avg_rpkm <- mean(expressed_values, na.rm = TRUE)

# Calculate the overall median RPKM for expressed genes
overall_median_rpkm <- median(expressed_values, na.rm = TRUE)

# Combining the results into a data frame
stats <- data.frame(
```

```
    Subset = name,
    TotalGenes = total_genes,
    ExpressedGenes = expressed_genes,
    NeverExpressedGenes = never_expressed_genes,
    PercentExpressed = percent_expressed,
    PercentNeverExpressed = percent_never_expressed,
    OverallAverageRPKM = overall_avg_rpkm,
    OverallMedianRPKM = overall_median_rpkm
  )

  return(stats)
}

# Applying the function to each subset of the data
# Each line creates a subset of the data and calculates the statistics for that subset
all_stats <- calculate_expression_stats(data, "All")
ice_stats <- calculate_expression_stats(data %>% filter(ENV == "ice"), "Ice")
cryo_stats <- calculate_expression_stats(data %>% filter(ENV == "cryoconite"), "Cryoconite")
prok_stats <- calculate_expression_stats(data %>% select(contains("PROK")), "PROK")
euk_stats <- calculate_expression_stats(data %>% select(contains("EUK")), "EUK")
ice_prok_stats <- calculate_expression_stats(data %>% filter(ENV == "ice") %>% select(contains("PROK")), "Ice + PROK")
ice_euk_stats <- calculate_expression_stats(data %>% filter(ENV == "ice") %>% select(contains("EUK")), "Ice + EUK")
cryo_prok_stats <- calculate_expression_stats(data %>% filter(ENV == "cryoconite") %>% select(contains("PROK")), "Cryoconite + PROK")
```

```
cryo_euk_stats <- calculate_expression_stats(data %>% filter(ENV == "cryoconite") %>%
select(contains("EUK")), "Cryoconite + EUK")


# Combining all statistics into one data frame

final_stats <- rbind(all_stats, ice_stats, cryo_stats, prok_stats, euk_stats,

                     ice_prok_stats, ice_euk_stats, cryo_prok_stats, cryo_euk_stats)


# Writing the combined statistics to a CSV file

write.csv(final_stats, "combined_expression_stats.csv", row.names = FALSE)
```

```
#################################################################################
####

#######      NMDS #################################################
```

```
library(tidyverse)

library(phyloseq)

library(microbiome)

library(vegan)

library(rwantshue)



# Define the get_wants_hue function

get_wants_hue <- function(n, seed = 1, palette = "colorblind_friendly"){

 if (n <= 0) {

  return(c())

 }

 scheme <- rwantshue::iwanthue(seed = seed, force_init = TRUE)

 scheme$hex(n, color_space = rwantshue::hcl_presets[[palette]])

}



##### STEP 1:
```

```
##ICE###

##rRNA##

ICE_rRNA_COUNTS_CSV <- "STOICice_COUNTS.csv"

ICE_rRNA_TAXA_CSV <- "STOICice_TAXAs.csv"


ice_rRNA_counts <- read_csv(ICE_rRNA_COUNTS_CSV) |>

  phyloseq::otu_table(taxa_are_rows = TRUE)


ice_rRNA_taxa <- readr::read_delim(ICE_rRNA_TAXA_CSV, delim = ";") |>

  as.matrix() |>

  phyloseq::tax_table()


## Reading ICEmetadata table

ice_MD_CSV <- read_csv("STOICice_METADATA 2.csv",

                   col_types = cols(DAYOFYEAR = col_character()))


#MD

icesiteMD <- ice_MD_CSV %>%

  tibble::column_to_rownames(var = "SEQUENCEFILE")|>

  phyloseq::sample_data()




##CRYOCONITE###


##rRNA##

CC_rRNA_COUNTS_CSV <- "STOICcc_COUNTS.csv"
```

```
CC_rRNA_TAXA_CSV <- "STOICcc_TAXA.csv"


cc_rRNA_counts <- read_csv(CC_rRNA_COUNTS_CSV) |>
  phyloseq::otu_table(taxa_are_rows = TRUE)


cc_rRNA_taxa <- readr::read_delim(CC_rRNA_TAXA_CSV, delim = ";") |>
  as.matrix() |>
  phyloseq::tax_table()



## Reading CCmetadata table
cc_MD_CSV <- read_csv("STOICcc_METADATA 2.csv",
           col_types = cols(DAYOFYEAR = col_character()))


#siteMD
ccsiteMD <- cc_MD_CSV %>%
  tibble::column_to_rownames(var = "SEQUENCEFILE")|>
  phyloseq::sample_data()


##### STEP 2:
#Make phyloseq objects


PHY_ice_rRNA_siteMD <- phyloseq(ice_rRNA_counts, ice_rRNA_taxa, icesiteMD)



PHY_cc_rRNA_siteMD <- phyloseq(cc_rRNA_counts, cc_rRNA_taxa, ccsiteMD)
```

```
#Remove site 6 from ice samples.

PHY_ice_rRNA_siteMD_site6 <- subset_samples(PHY_ice_rRNA_siteMD, SITE != "6")



##### STEP 3:

###NMDS


##Cryoconite

CC_physeq_nmds <- ordinate(PHY_cc_rRNA_siteMD, method = "NMDS", distance = "bray")


## ICE

ICE_physeq_nmds <- ordinate(PHY_ice_rRNA_siteMD_site6, method = "NMDS", distance =
"bray")


##### STEP 4:

# Create NMDS plot



##Cryoconite


NMDS_CC_rRNA <- plot_ordination(PHY_cc_rRNA_siteMD, CC_physeq_nmds, color
="DAYOFYEAR", shape = "NAME") +

  geom_point(size=3) +

  scale_color_manual(values=get_wants_hue(7)) +
```

```
 theme(legend.title = element_blank()) +
 theme(strip.background = element_rect(fill="white")) +
 theme_bw() +
 ggtitle("rRNA abundance in Cryoconite")  + labs(shape = "Site")

NMDS_CC_rRNA




#ICE
NMDS_ICE_rRNA <- plot_ordination(PHY_ice_rRNA_siteMD_site6, ICE_physeq_nmds, color = as.character("DAYOFYEAR"), shape = "NAME") +
 geom_point(size=3) +
 scale_color_manual(values=get_wants_hue(7)) +
 theme(legend.title = element_blank()) +
 theme(strip.background = element_rect(fill="white")) +
 theme_bw() +
 ggtitle("rRNA abundance in Ice")  + labs(shape = "Site")

NMDS_ICE_rRNA




##########################################################################################################
```

```
################ BGC expression NMDS
####################################################

# filtered expression data to only include BGCs expressed in over half of samples.



##### STEP 1:

##ICE###

##rRNA##

ICE_BGC_COUNTS_CSV <- "ice_BGC_exp_50.csv"

ICE_BGC_TAXA_CSV <- "ice_BGC_tax_50.csv"

ice_BGC_counts <- read_csv(ICE_BGC_COUNTS_CSV) |>

  phyloseq::otu_table(taxa_are_rows = TRUE)

ice_BGC_taxa <- readr::read_delim(ICE_BGC_TAXA_CSV, delim = ";") |>

  as.matrix() |>

  phyloseq::tax_table()

## Reading ICEmetadata table

ice_MD_CSV <- read_csv("ice_BGC_meta.csv",

                   col_types = cols(DAYOFYEAR = col_character()))

#MD

icesiteMD <- ice_MD_CSV %>%
```

```
  tibble::column_to_rownames(var = "SEQUENCEFILE")|>
  phyloseq::sample_data()



##CRYOCONITE###


##rRNA##
CC_BGC_COUNTS_CSV <- "cc_BGC_exp_50.csv"
CC_BGC_TAXA_CSV <- "cc_BGC_tax_50.csv"


cc_BGC_counts <- read_csv(CC_BGC_COUNTS_CSV) |>
  phyloseq::otu_table(taxa_are_rows = TRUE)


cc_BGC_taxa <- readr::read_delim(CC_BGC_TAXA_CSV, delim = ";") |>
  as.matrix() |>
  phyloseq::tax_table()



## Reading CCmetadata table
cc_MD_CSV <- read_csv("cc_BGC_meta.csv",
                      col_types = cols(DAYOFYEAR = col_character()))


#siteMD
ccsiteMD <- cc_MD_CSV %>%
  tibble::column_to_rownames(var = "SEQUENCEFILE")|>
  phyloseq::sample_data()
```

```
##### STEP 2:

#Make phyloseq objects


PHY_ice_BGC_siteMD <- phyloseq(ice_BGC_counts, ice_BGC_taxa, icesiteMD)



PHY_cc_BGC_siteMD <- phyloseq(cc_BGC_counts, cc_BGC_taxa, ccsiteMD)





##### STEP 3:

###NMDS


##Cryoconite

CC_physeq_nmds <- ordinate(PHY_cc_BGC_siteMD, method = "NMDS", distance = "bray")


## ICE

ICE_physeq_nmds <- ordinate(PHY_ice_BGC_siteMD, method = "NMDS", distance = "bray")


##### STEP 4:

# Create NMDS plot
```

```
##Cryoconite

NMDS_CC_BGC <- plot_ordination(PHY_cc_BGC_siteMD, CC_physeq_nmds, color
="DAYOFYEAR", shape = "NAME") +
  geom_point(size=3) +
  scale_color_manual(values=get_wants_hue(7)) +
  theme(legend.title = element_blank()) +
  theme(strip.background = element_rect(fill="white")) +
  theme_bw() +
  ggtitle("BGC expression in Cryoconite")  + labs(shape = "Site")

NMDS_CC_BGC

#ICE
NMDS_ICE_BGC <- plot_ordination(PHY_ice_BGC_siteMD, ICE_physeq_nmds, color =
as.character("DAYOFYEAR"), shape = "NAME") +
  geom_point(size=3) +
  scale_color_manual(values=get_wants_hue(7)) +
  theme(legend.title = element_blank()) +
  theme(strip.background = element_rect(fill="white")) +
  theme_bw()+
  ggtitle("BGC expression in Ice")  + labs(shape = "Site")
```

NMDS_ICE_BGC
