# Supplementary figures and images for: The encoded and expressed biosynthetic potential of Greenland Ice Sheet microbes
Source: Front Microbiol. 2025 Jul 31;16:1620548. doi: 10.3389/fmicb.2025.1620548 (PMC12350317; doi:10.3389/fmicb.2025.1620548)

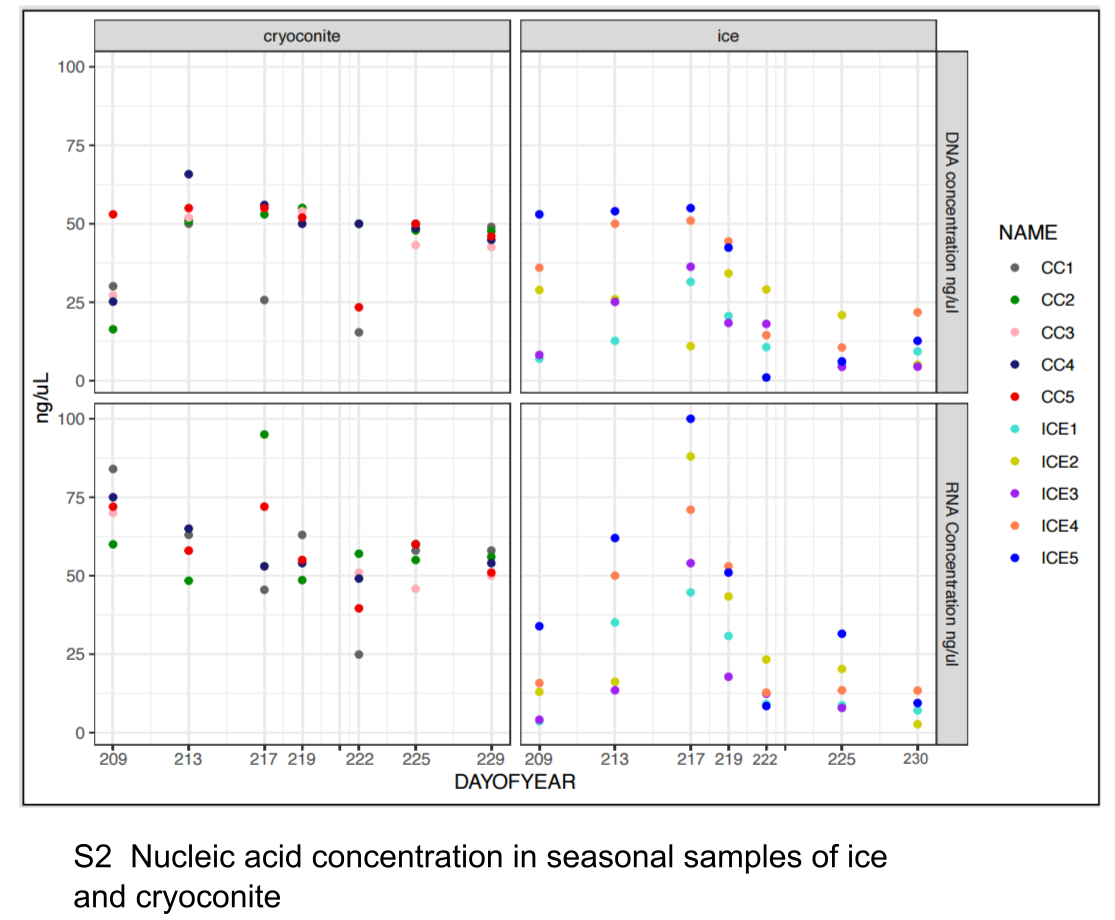

Supplement: Supplementary file 4 [file Image_1.png]

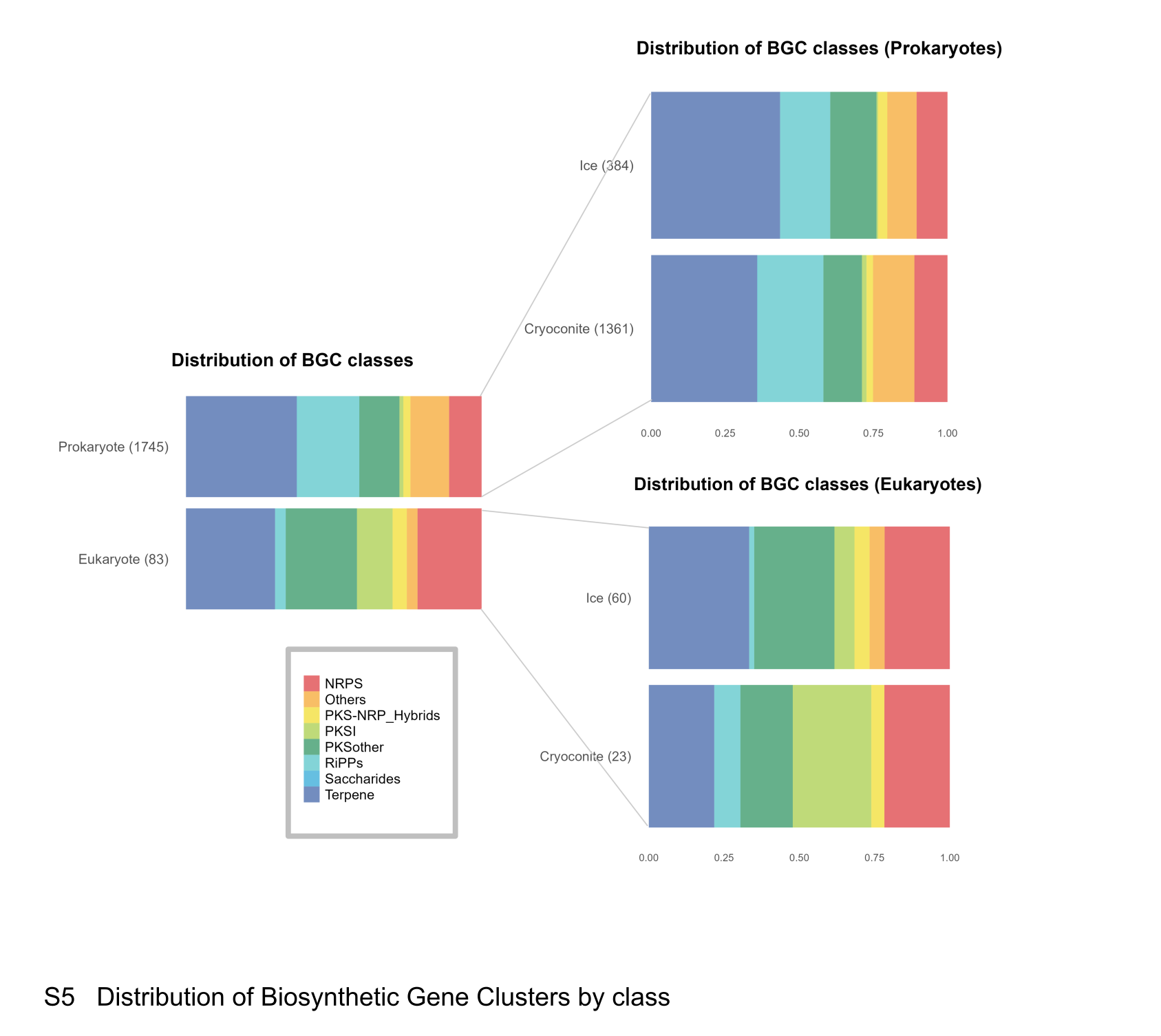

Supplement: Supplementary file 5 [file Image_2.png]

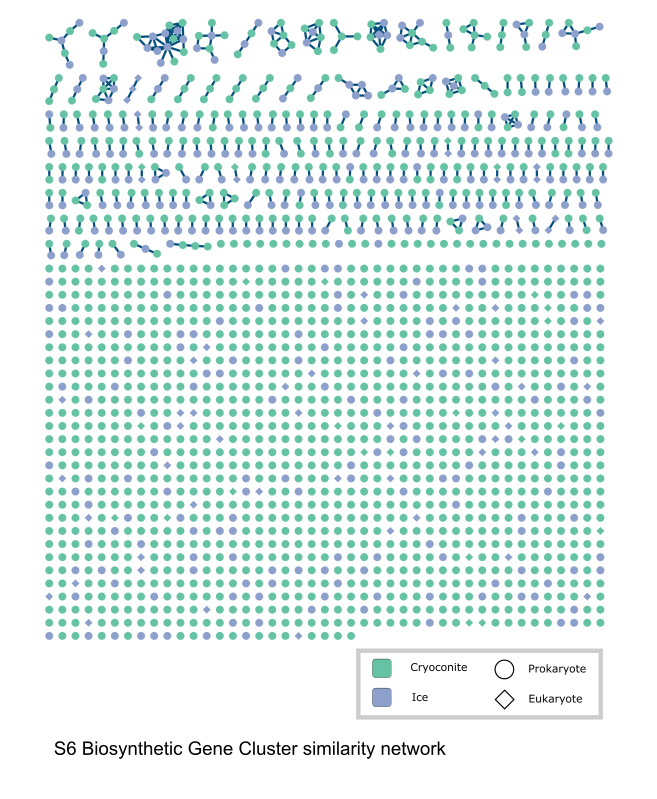

Supplement: Supplementary file 6 [file Image_3.png]

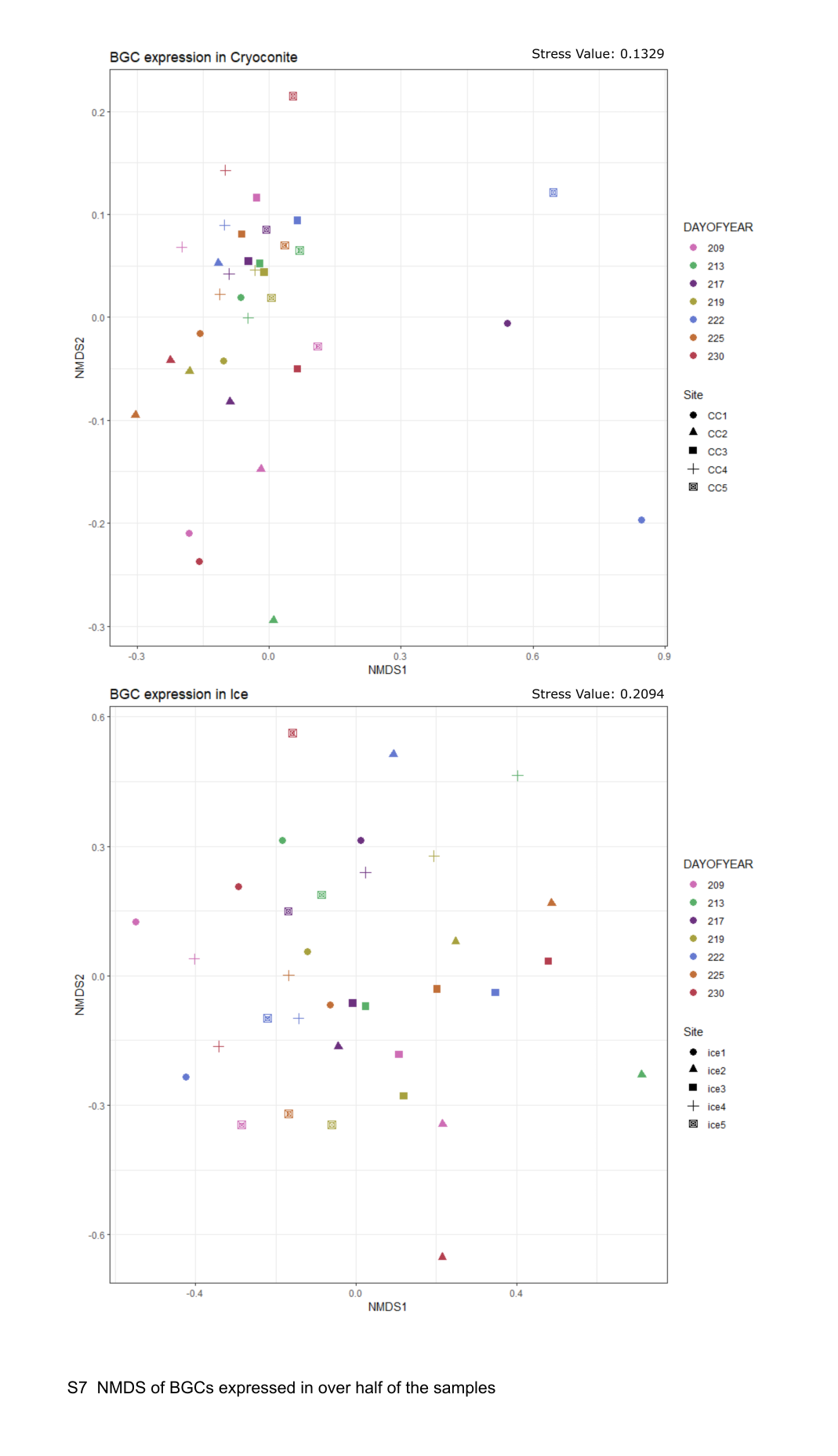

Supplement: Supplementary file 7 [file Image_4.png]

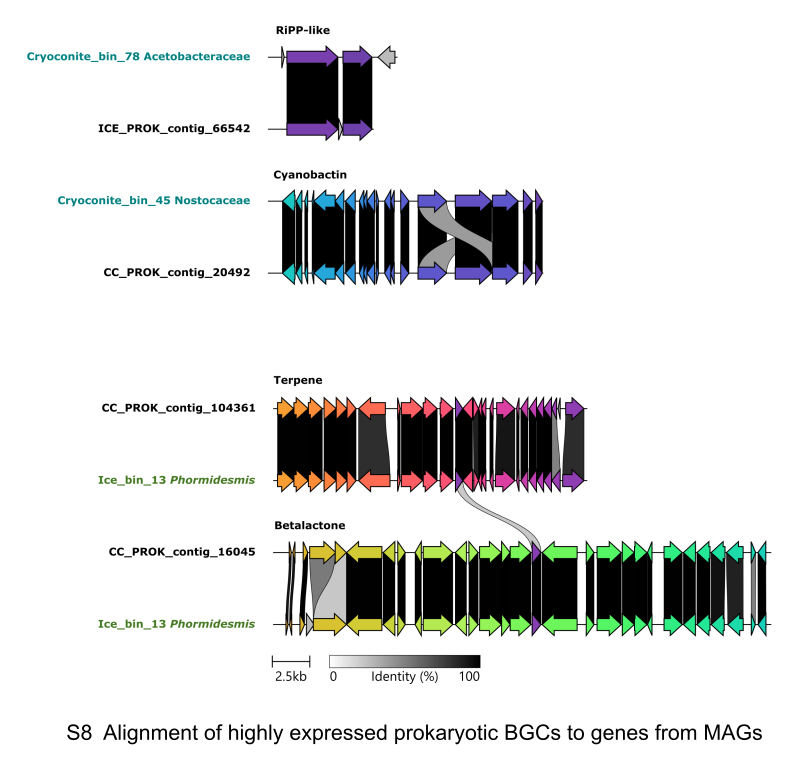

Supplement: Supplementary file 8 [file Image_5.png]
